# Efficient donor–acceptor host materials for green organic light-emitting devices: non-doped blue-emissive materials with dual charge transport properties†

**DOI:** 10.1039/c8ra02840k

**Published:** 2018-05-31

**Authors:** Jayaraman Jayabharathi, Palanisamy Sujatha, Venugopal Thanikachalam, Pavadai Nethaji

**Affiliations:** Department of Chemistry, Annamalai University Annamalainagar 608 002 Tamilnadu India jtchalam2005@yahoo.co.in +91 9443940735

## Abstract

Comparative optical, electroluminescence and theoretical studies were performed for (*E*)-4′-(1-(4-(2-(1-(4-morpholinophenyl)-1*H*-phenanthro[9,10-*d*]imidazol-2-yl)vinyl)phenyl)-1*H*-phenanthro[9,10-*d*]imidazol-2-yl)-*N*,*N*-diphenyl-[1,1′-biphenyl]-4-amine (SMPI-TPA) and (*E*)-4-(4-(2-(4-(2-(4-(9*H*-carbazol-9-yl)phenyl)-1*H*-phenanthro[9,10-*d*]imidazol-1-yl)styryl)-1*H*-phenanthro[9,10-*d*]imidazol-1-yl)phenyl)morpholine (SMPI-Cz). These compounds show excellent thermal properties, dual charge transport properties and form thin films under thermal evaporation. Blue OLEDs (CIE: 0.16, 0.08) based on SMPI-TPA show efficient device performance (*η*_ex_ 6.1%; *η*_c_ 5.3 cd A^−1^; *η*_p_ 5.2 lm W^−1^) at low turn-on voltages. Both SMPI-TPA and SMPI-Cz were utilised as hosts for green OLEDs. The devices with SMPI-Cz (30 nm):5 wt% Ir(ppy)_3_ exhibit maximum luminance of 20 725 cd m^−2^, and *η*_c_ and *η*_p_ values of 61.4 cd A^−1^ and 63.8 lm W^−1^, respectively. In comparison, devices with SMPI-TPA (30 nm):5 wt% Ir(ppy)_3_ exhibit high *η*_c_ and *η*_p_ values of 65.2 cd A^−1^ and 67.1 lm W^−1^, respectively. Maximum *η*_ex_ values of 19.6% and 23.4% were obtained from SMPI-TPA:Ir(ppy)_3_ and SMPI-Cz:Ir(ppy)_3_, respectively. These device performances indicate that the phenanthroimidazole unit is a tunable building unit for efficient carrier injection and it may also be employed as a host for green OLEDs.

## Abstract 2

SMPI-Cz:Ir(ppy)_3_-based devices exhibit a luminance of 20 725 cd m^−2^, *η*_c_ of 61.4 cd A^−1^ and *η*_p_ of 63.8 lm W^−1^.

## Graphical abstract

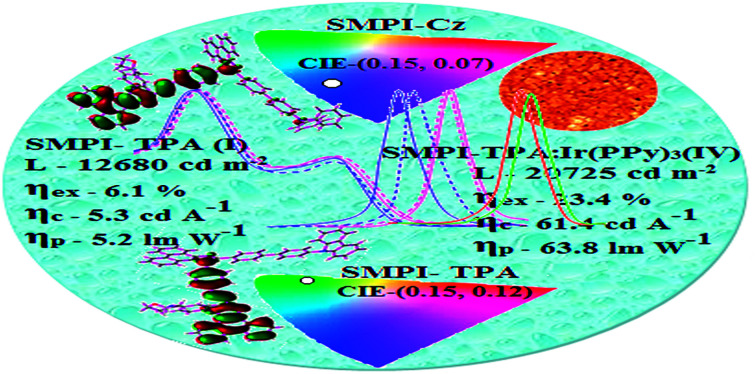

## 1. Introduction

Efficient green or red OLEDs with pure color CIE coordinates have been reported^1–4^ and blue emitters with less power consumption in organic optoelectronics have also been broadly studied.^5,6^ However, there is need for long-lifetime blue emitters with pure colour CIE coordinates due to wide band gaps (*E*_g_), which require limited π-conjugation length.^7–9^ Simultaneous carrier injection into a blue-emissive layer becomes very difficult due to its wide *E*_g_, resulting in a decrease in device efficiency.^10,11^ Therefore, for OLED applications, highly efficient and low-cost blue OLEDs are of urgent demand. The external quantum efficiency (*η*_ex_) can be deduced from the following equation: *η*_ex_ = *η*_IQE_ × *η*_out_ = *η*_rec_ × *η*_PL_ × *η*_S_ × *η*_out_, where *η*_IQE_ is the internal quantum efficiency, *η*_out_ (∼1/2*n*^2^) is the light out-coupling efficiency (*n* = 1.5, *η*_out_ ∼ 20%), *η*_rec_ is the electron–hole recombination efficiency (100%), *η*_PL_ is the photoluminescence efficiency and *η*_S_ is the utilization efficiency. The two key parameters *η*_PL_ and *η*_S_ required for high *η*_ex_ can be tuned by altering the emitter molecular design.^12^

A blue-emissive material with high triplet energy (*E*_T_) may be employed as a host for green OLEDs.^13–16^ However, the high triplet energy enables green emitters to harvest the triplet energy of a blue emitter; also, efficient non-doped blue emitters are not suitable as hosts for phosphorescent OLEDs due to their low *E*_T_ as well as poor carrier transport properties.^17^ Moreover, efficient hosts for green emitter exhibit low efficiency when they are used as emissive materials in blue OLEDs.^18–20^ Hence, efforts are still required to achieve efficient OLEDs based on blue emissive materials.

Therefore, to develop dual-functional emissive materials, *i.e.*, emitters for blue OLEDs and hosts for green OLEDs, synthesis of molecules with donor (D)/acceptor (A) (electron/hole transport moieties) configuration has gained interest. To achieve deep blue emission, the donor–acceptor (D–A) molecule should have relatively weak charge transfer properties since a strong D–A geometry can induce red-shifted emission. In addition, the singlet–triplet splitting should be small to ensure that the triplet-excited state energy is high enough to excite the green phosphorescent dopant.

With our continuous interest to synthesize efficient n-type imidazole derivatives as OLED emitters,^21–23^ herein we report the synthesis of (*E*)-4′-(1-(4-(2-(1-(4-morpholinophenyl)-1*H*-phenanthro[9,10-*d*]imidazol-2-yl)vinyl)phenyl)-1*H*-phenanthro[9,10-*d*]imidazol-2-yl)-*N*,*N*-diphenyl-[1,1′-biphenyl]-4-amine (SMPI-TPA) and (*E*)-4-(4-(2-(4-(2-(4-(9*H*-carbazol-9-yl)phenyl)-1*H*-phenanthro[9,10-*d*]imidazol-1-yl)styryl)-1*H*-phenanthro[9,10-*d*]imidazol-1-yl)phenyl)morpholine (SMPI-Cz) derivatives and utilize them as blue emitters in non-doped devices and hosts for green OLEDs. These derivatives exhibit higher stability with balanced injection property, leading to excellent device performances.

## 2. Materials and measurements

The structure of emissive materials was confirmed by ^1^H and ^13^C NMR spectroscopies and mass spectrometry, recorded using a Bruker spectrometer (400 MHz) and Agilent (LCMS VL SD), respectively. Redox potentials were measured using a potentiostat CHI 630A electrochemical analyzer. The Lambda 35 PerkinElmer instrument spectrophotometer with integrated sphere (RSA-PE-20) was used to measure absorbance in both solution and film states. Emissive properties (PL) were analyzed *via* PerkinElmer LS55 fluorescence spectrometer measurements. Thermal characteristics such as decomposition (*T*_d_) and glass transition (*T*_g_) temperatures were analyzed using a PerkinElmer thermal analysis system and NETZSCH-DSC-204, respectively, with heating rate of 10 °C min^−1^ under N_2_ atmosphere. Lifetime measurements of SMPI-TPA and SMPI-Cz were recorded using a time-correlated single-photon counting spectrometer (TCSPC: Horiba Fluorocube-01-NL lifetime system). The *Φ* (PL quantum yield) was measured in dichloromethane using 0.5 M H_2_SO_4_:quinine (0.54) as reference.

### 2.1. Theoretical calculations

Using the Gaussian 09 software package,^24^ the electron density on frontier molecular orbitals of SMPI-TPA and SMPI-Cz was identified.

### 2.2. Devices fabrication

OLEDs with SMPI-TPA and SMPI-Cz emitters were fabricated by vacuum deposition at 5 × 10^−6^ Torr on a precleaned ITO substrate with 20 Ω per square resistance (rate −0.1 nm s^−1^). LiF and Al were also evaporated thermally on the organic layer and the thickness was measured using a quartz crystal thickness monitor. A series of fabricated devices with multilayer configuration are denoted as follows: ITO/NPB (4,4′-bis[*N*-(1-naphthyl)-*N*-phenylamino]biphenyl) (50 nm)/TCTA (tris(4-carbazoyl-9-ylphenyl)amine) (5 nm)/SMPI-TPA (I) or SMPI-Cz (II) (30 nm)/TPBI (2,2′,2′′-(1,3,5-benzinetriyl)-tris(1-phenyl-1-*H*-benzimidazole)) (15 nm)/LiF (1 nm)/Al (100 nm); ITO/NPB (50 nm)/SMPI-TPA (30 nm):5 wt% Ir(PPy)_3_ or (III) or SMPI-Cz (30 nm):5 wt% Ir(PPy)_3_ (IV)/BCP (2,9-dimethyl-4,7-diphenyl-1,10-phenanthroline) (15 nm)/Alq_3_ (50 nm)/LiF (1 nm)/Al (100 nm). Further, single-carrier devices were also been fabricated. The hole-only device has the configuration of ITO/NPB (50 nm)/SMPI-TPA or SMPI-Cz (30 nm)/NPB (50 nm)/Al (200 nm), while the electron-only device has the configuration of ITO/TPBI (20 nm) SMPI-TPA or SMPI-Cz (30 nm)/TPBI (20 nm)/LiF (1 nm)/Al (200 nm). Electrical measurements were recorded using a Keithley 2400 sourcemeter.

#### 2.2.1. Synthesis of 2-(4-nitrostyryl)-1-(4-morpholinophenyl)-1*H*-phenanthro[9,10-*d*]imidazole

Phenanthrenequinone (2.08 g, 10 mmol), 4-nitrocinnamaldehyde (1.51 g, 10 mmol), 4-morpholinobenzenamine (4.65 g, 50 mmol) and ammonium acetate (3.08 g, 40 mmol), all in acetic acid (25 mL), were refluxed (120 °C; 12 h; N_2_ stream). Then, 2-(4-nitrostyryl)-1-(4-morpholinophenyl)-1*H*-phenanthro[9,10-*d*]imidazole was purified and used for further analyses (Scheme S1†). Anal. calcd C_33_H_26_N_4_O_3_: C, 75.26; H, 4.88; N, 10.67. Found: C, 75.23; H, 4.86; N, 10.65. ^1^H NMR (400 MHz, CDCl_3_): *δ* 2.97 (s, 4H), 3.58 (s, 4H), 6.54 (d, *J* = 8.8 Hz, 2H), 7.13 (d, *J* = 16.0 Hz, 1H), 7.18 (d, *J* = 15.2 Hz, 1H), 7.32 (s, 2H), 7.52 (d, *J* = 8.6 Hz, 2H), 7.71–7.83 (m, 6H), 8.02 (s, 1H), 8.13 (d, *J* = 8.4 Hz, 2H), 8.51 (d, *J* = 7.2 Hz, 1H).·^13^C NMR (100 MHz, CDCl_3_): *δ* 47.33, 62.42, 114.68, 119.43, 119.86, 120.31, 120.78, 121.12, 121.44, 122.85, 123.12, 123.85, 124.02, 124.70, 124.85, 126.17, 125.26, 125.39, 126.67, 126.19, 126.44, 126.53, 126.74, 129.72, 131.93, 133.45, 146.67. MS: *m*/*z* 526.58 [M+]; calcd 526.62.

#### 2.2.2. Synthesis of 4-((*E*)-2-(1-(4-morpholinophenyl)-1H-phenanthro[9,10-*d*]imidazol-2-yl)vinyl)benzenamine

Initially, 2-(4-nitrostyryl)-1-(4-morpholinophenyl)-1*H*-phenanthro[9,10-*d*]imidazole (4.15 g, 10 mmol) and 10% Sn/HCl (250 mg) in 25 mL ethanol were refluxed under stirring. Then, 80% hydrazine hydrate (15 mL) was added, and stirring was continued for one day. The solution was treated with water : HCl mixture, and the obtained white product was used after purification. Anal. calcd C_33_H_28_N_4_O: C, 79.87; H, 5.62; N, 11.18. Found: C, 79.82; H, 5.60; N, 11.15. ^1^H NMR (400 MHz, CDCl_3_): *δ* 2.72 (s, 4H), 3.36 (s, 4H), 4.12 (s, 2H), 6.43 (s, 1H), 6.93 (d, *J* = 16.2 Hz, 1H), 7.06 (d, *J* = 15.4 Hz, 1H), 7.23 (s, 2H), 7.36 (d, *J* = 8.6 Hz, 3H), 7.59–7.68 (m, 5H), 7.92 (s, 2H), 8.03 (d, *J* = 8.6 Hz, 2H), 8.62 (d, *J* = 7.2 Hz, 1H). ^13^C NMR (100 MHz, CDCl_3_): *δ* 42.28, 57.36, 112.61, 120.18, 120.76, 121.121, 121.43, 122.28, 122.67, 123.73, 124.08, 124.65, 125.12, 125.56, 125.92, 126.28, 126.32, 126.49, 127.52, 127.63, 127.82, 128.27, 128.56, 130.63, 132.53, 133.06, 145.27. MS: *m*/*z* 496.65 [M+]; calcd 496.60.

#### 2.2.3. Synthesis of (*E*)-4′-(1-(4-(2-(1-(4-morpholinophenyl)-1*H*-phenanthro[9,10-*d*]imidazol-2-yl)vinyl)phenyl)-1*H*-phenanthro[9,10-*d*]imidazol-2-yl)-*N*,*N*-diphenyl-[1,1′-biphenyl]-4-amine (SMPI-TPA)

Phenanthrenequinone (0.416 g, 2 mmol), 4′-(diphenylamino)biphenyl-4-carbaldehyde (0.698 g, 2 mmol), 4-((*E*)-2-(1-(4-morpholinophenyl)-1*H*-phenanthro[9,10-*d*]imidazol-2-yl)vinyl)benzenamine (1.16 g, 3 mmol) and ammonium acetate (1.54 g, 20 mmol), all in 25 mL acetic acid, were refluxed (120 °C; 12 h; N_2_ stream), and the obtained gray white solid was used for further analyses after purification. Anal. calcd C_72_H_52_N_6_O: C, 85.07; H, 5.17; N, 8.28. Found: C, 85.01; H, 5.11; N, 8.25. ^1^H NMR (400 MHz, CDCl_3_): *δ* 3.12 (s, 4H), 3.56 (s, 4H), 6.58 (s, 4H), 6.64–6.72 (m, 6H), 6.82 (s, 1H), 6.94 (d, *J* = 16.2 Hz, 1H), 7.24–7.38 (m, 9H), 7.48 (s, 2H), 7.56 (d, *J* = 8.6 Hz, 2H), 7.62 (s, 2H), 7.72–7.96 (m, 14H), 8.28 (d, *J* = 7.6 Hz, 2H), 8.78 (d, *J* = 16.2 Hz, 1H). ^13^C NMR (100 MHz, CDCl_3_): *δ* 52.81, 65.36, 112.82, 117.45, 122.42, 122.66, 122.92, 125.58, 125.82, 126.26, 126.59, 126.78, 126.92, 127.21, 127.83, 128.62, 128.89, 130.17, 130.43, 133.48, 137.48, 138.26, 140.62, 144.51, 149.68. MS: *m*/*z* 1016.45 [M+]; calcd 1016.40.

#### 2.2.4. Synthesis of (*E*)-4-(4-(2-(4-(2-(4-(9*H*-carbazol-9-yl)phenyl)-1*H*-phenanthro[9,10-*d*]imidazol-1-yl)styryl)-1*H*-phenanthro[9,10-*d*]imidazol-1-yl)phenyl)morpholine (SMPI-Cz)

Phenanthrenequinone (0.416 g, 2 mmol), 4-(9*H*-carbazol-9-yl)benzaldehyde (0.698 g, 2 mmol), 4-((*E*)-2-(1-(4-morpholinophenyl)-1*H*-phenanthro[9,10-*d*]imidazol-2-yl)vinyl)benzenamine (1.16 g, 3 mmol) and ammonium acetate (1.54 g, 20 mmol), all in 25 mL glacial acetic acid, were refluxed (120 °C; 12 h; N_2_ stream), and the obtained gray white solid was used for further analyses after purification. Anal. calcd C_66_H_46_N_6_O: C, 84.47; H, 4.92; N, 8.95. Found: C, 84.42; H, 4.89; N, 8.91. ^1^H NMR (400 MHz, CDCl_3_): *δ* 2.96 (s, 4H), 3.38 (s, 4H), 6.66 (s, 2H), 6.86 (d, *J* = 16.4 Hz, 1H), 6.98 (s, 1H), 7.18 (d, *J* = 8.6 Hz, 1H), 7.32–7.42 (m, 11H), 7.68 (d, *J* = 8.6 Hz, 2H), 7.82 (s, 2H), 7.96–8.24 (m, 13H), 8.48 (d, *J* = 7.6 Hz, 2H), 8.92 (d, *J* = 16.2 Hz, 1H). ^13^C NMR (100 MHz, CDCl_3_): *δ* 49.42, 63.34, 109.48, 112.73, 114.23, 117.82, 121.38, 121.67, 122.48, 122.73, 122.98, 124.62, 125.34, 125.74, 126.18, 126.48, 126.67, 126.93, 127.74, 128.53, 128.77, 130.34, 133.52, 137.84, 138.43, 140.89, 144.68, 150.51. MS: *m*/*z* 938.42 [M+]; calcd 938.40.

## 3. Results and discussion

As depicted in Scheme S1,† the blue emitters used as hosts for green OLEDs, namely, (*E*)-4′-(1-(4-(2-(1-(4-morpholinophenyl)-1*H*-phenanthro[9,10-*d*]imidazol-2-yl)vinyl)phenyl)-1*H*-phenanthro[9,10-*d*]imidazol-2-yl)-*N*,*N*-diphenyl-[1,1′-biphenyl]-4-amine (SMPI-TPA) and (*E*)-4-(4-(2-(4-(2-(4-(9*H*-carbazol-9-yl)phenyl)-1*H*-phenanthro[9,10-*d*] imidazol-1-yl)styryl)-1*H*-phenanthro[9,10-*d*]imidazol-1-yl)phenyl)morpholine (SMPI-Cz) were prepared from phenanthrenequinone (0.416 g, 2 mmol), 4′-(diphenylamino)biphenyl-4-carbaldehyde (SMPI-TPA)/4-(9*H*-carbazol-9-yl)benzaldehyde (SMPI-Cz), 4-((*E*)-2-(1-(4-morpholinophenyl)-1*H*-phenanthro[9,10-*d*]imidazol-2-yl)vinyl)benzenamine and ammonium acetate in acetic acid (120 °C; 12 h; N_2_ stream). The formed SMPI-TPA and SMPI-Cz were analysed by various spectroscopic methods, from which their molecular structures were confirmed.

### 3.1. Potential energy surface (PES) scan studies and thermal properties

**Fig. 1:**
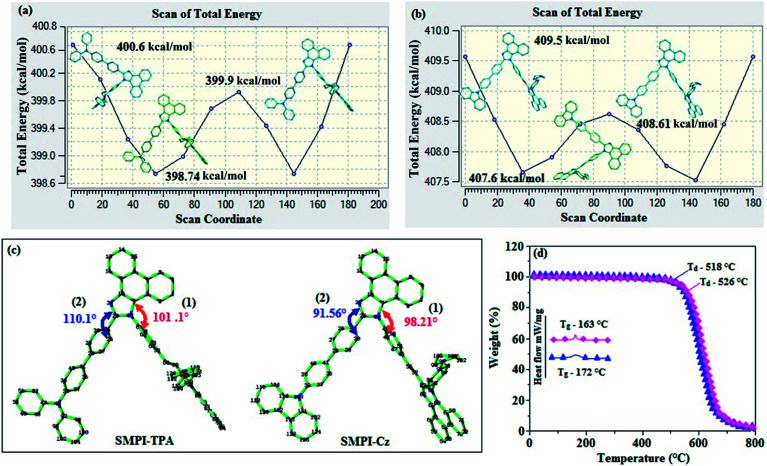
Potential energy scan diagram of (a) SMPI-TPA and (b) SMPI-Cz; (c) dihedral angle of SMPI-Cz: (1) C(7)–N(23)–C(49)–C(48); (2) C(24)–N(25)–C(31)–C(30) and SMPI-TPA: (1) C(7)–N(23)–C(61)–C(66); (2) N(24)–C(25)–C(31)–C(26); (d) DSC and TGA of SMPI-TPA and SMPI-Cz.

The potential energy surface scan of N24–C25–C31–C30 (SMPI-Cz)/N24–C25–C31–C26 (SMPI-TPA) was performed using DFT/B3LYP/6-31G (d,p). During calculation, all the geometrical parameters were simultaneously relaxed, while their respective torsional angles were varied in steps of 0°, 20°, 40°, 60°…360°. The potential energy surface diagrams (Fig. 1a and b) reveal that the minimum energy conformation corresponds to that in which the 4-morpholinophenyl ring attached to the imidazole nitrogen atom (N23) is tilted to an angle of 98.21° (SMPI-Cz)/101.1° (SMPI-TPA) and the phenyl ring with carbazole core (SMPI-Cz) and another phenyl ring with triphenylamine core (SMPI-TPA) are attached to the imidazole carbon atom (C25) at an angle of 91.56° (SMPI-Cz)/110.1° (SMPI-TPA) (Fig. 1c). Owing to rigid molecular back bone and non-coplanar geometry, these compounds (SMPI-TPA and SMPI-Cz) exhibit suitable thermal stability [high decomposition (*T*_d5_)/high glass transition (*T*_g_) temperature: 526/163 °C-SMPI-TPA and 518/172 °C-SMPI-Cz] (Fig. 1d; Table 1). The high *T*_g_ and *T*_d5_ values improve the lifetime of the OLEDs by forming thin films upon vacuum evaporation.^21^

**Table 1:** Optical and thermal properties and device performances of SMPI-TPA, SMPI-Cz, SMPI-TPA:Ir(PPy)_3_ and SMPI-Cz:Ir(PPy)_3_

Emitters	SMPI-TPA (I)	SMPI-Cz (II)	SMPI-TPA:Ir(PPy)_3_ (III)	SMPI-Cz:Ir(PPy)_3_ (IV)
**Optical and thermal properties**				
*λ*_ab_ (nm) (asol/bfilm)	250, 358/252, 360	248, 355/250, 357	—	—
*λ*_em_ (nm) (asol/bfilm)	441/439	412/401	—	—
*T*_g_/*T*_d_ (°C)	163/526	172/518	—	—
PLQY^c^ (asol/bfilm)	0.81/0.56	0.69/0.71	—	—
HOMO/LUMO^d^ (eV)	−5.41/−2.15	−5.39/−2.17	—	—

**Device efficiency**				
*L* (cd m^−2^)	12 680	12 468	1094	20 725
*V*_on_ (V)	3.1	3.2	2.8	2.9
*η*_ex_ (%)	6.1	5.9	19.6	23.4
*η*_c_ (cd A^−1^)	5.3	5.1	65.2	61.4
*η*_p_ (lm W^−1^)	5.2	4.6	67.1	63.8

a Measured in dilute toluene solution (×10^−5^ mol L^−1^) at room temperature.

b Measured neat film by coating.

c Absolute PL quantum yield evaluated using an integrating sphere.

d Measured by cyclic voltammetry calculated by comparing with ferrocene (Fc).

### 3.2. HOMO–LUMO

**Fig. 2:**
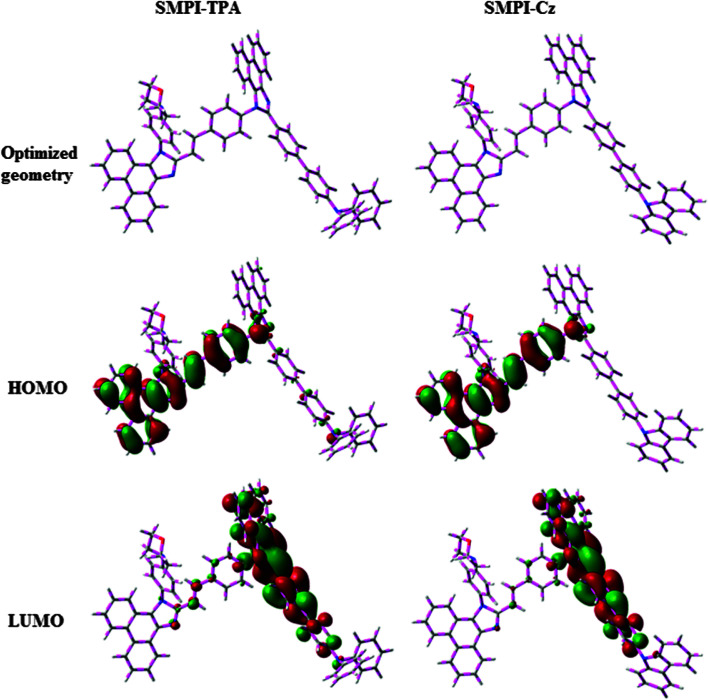
Optimised geometry, and HOMO and LUMO contour plots of SMPI-TPA and SMPI-Cz.

**Fig. 3:**
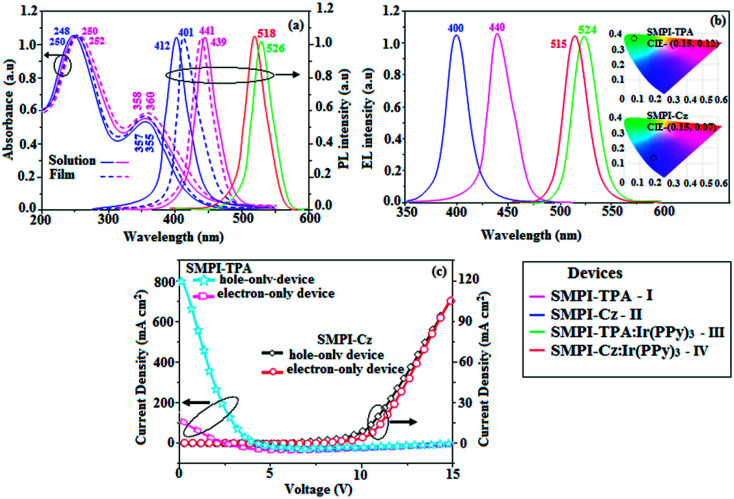
Normalized (a) absorption, emission and (b) EL spectra of SMPI-TPA, SMPI-Cz, SMPI-TPA:5 wt% Ir (PPy)_3_:SMPI-TPA film and SMPI-Cz:Ir (PPy)_3_:SMPI-Cz film and (c) hole-only and electron-only devices.

The HOMO of both SMPI-TPA and SMPI-Cz is localized on the fragment attached to phenanthrimidazole nitrogen, while the LUMO is distributed on the phenanthrimidazole with TPA (SMPI-TPA) and Cz (SMPI-Cz) fragments (Fig. 2). The HOMO and LUMO of SMPI-TPA and SMPI-Cz display adequate separation in electron density features, which enhances the hole- and electron-transport functions and also reduces the singlet–triplet splitting.^22^ Moreover, SMPI-TPA and SMPI-Cz exhibit redox waves, supporting their carrier transport abilities. The HOMO energies of −5.41 eV (SMPI-TPA) and −5.39 eV (SMPI-Cz) are determined from their respective oxidation onset potentials of 0.61 V (SMPI-TPA) and 0.59 V (SMPI-Cz) [*E*_HOMO_ = −(*E*_ox_ + 4.8 eV)].^23^ The LUMO energies of −2.15 eV (SMPI-TPA) and −2.17 eV (SMPI-Cz) are calculated using the equation *E*_LUMO_ = *E*_HOMO_ − 1239/*λ*_onset_. The charge-transporting properties of SMPI-TPA and SMPI-Cz were investigated by fabricating single-carrier devices. The hole-only device has the configuration of ITO/NPB (50 nm)/SMPI-TPA or SMPI-Cz (30 nm)/NPB (50 nm)/Al (200 nm) and the electron-only device has the configuration of ITO/TPBI (20 nm) SMPI-TPA or SMPI-Cz (30 nm)/TPBI(20 nm)/LiF (1 nm)/Al (200 nm). NPB on the cathode side of the hole-only device and TPBI (1,3,5-tri(phenyl-2-benzimidazolyl)benzene) on the anode side of the electron-only device were used to block electron injection from Al and hole injection from ITO, respectively. Fig. 3 shows that both devices can significantly conduct current, indicating that SMPI-TPA and SMPI-Cz are capable of transporting both holes and electrons and they exhibit a bipolar transporting nature. This is beneficial for balancing the holes and electrons in the emitting layer based on SMPI-TPA and SMPI-Cz.

### 3.3. Photophysical properties

**Fig. 4:**
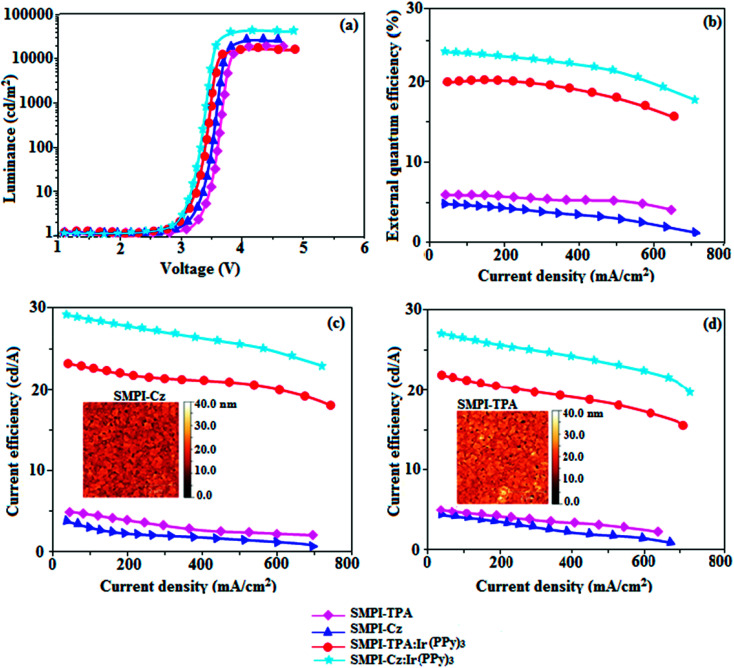
Electroluminescence performances: (a) luminance *versus* voltage; (b) external quantum efficiency *versus* current density; (c) current efficiency *versus* current density and (d) power efficiency *versus* current density of devices I–IV based on SMPI-TPA (I), SMPI-Cz (II), SMPI-TPA:Ir(PPy)_3_ (III) and SMPI-Cz:Ir(PPy)_3_ (IV), [inset: AFM images of SMPI-TPA film and SMPI-Cz film].

**Fig. 5:**
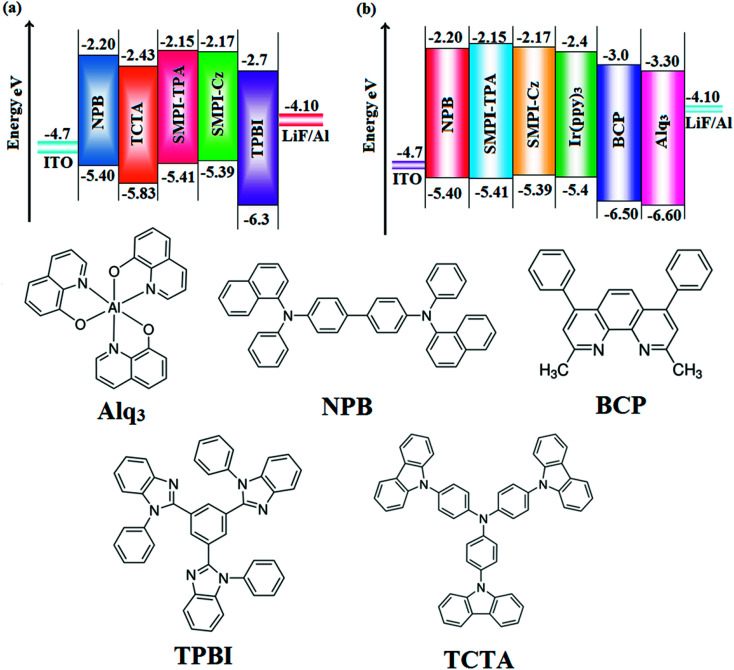
Energy-level diagram of (a) non-doped and (b) doped devices and the materials used for the fabrication of devices.

Electronic spectral studies of SMPI-TPA and SMPI-Cz were carried out in dichloromethane (Fig. 3). The results indicated that *λ*_abs_ at around 248 nm originated from the aryl group attached to the nitrogen of phenanthrimidazole plane, while *λ*_abs_ at around 355 nm was attributed to π → π* electronic transition of the styryl phenanthrimidazole ring. The origin of absorption of SMPI-TPA and SMPI-Cz was studied by computational methods. The outcomes of computational and experimental methods are similar [HOMO–LUMO+1 transition; Table S1(a) & (b)†]. These SMPI-TPA and SMPI-Cz derivatives show blue emission at 441 and 412 nm, respectively, and the measured PLQY values for SMPI-TPA and SMPI-Cz in solution/film are of 0.81/0.56 and 0.69/0.71, respectively. The emission maximum of SMPI-Cz (412 nm) is blue-shifted when compared with SMPI-TPA (441 nm) due to the additional phenyl ring in SMPI-TPA, which induces an elongated conjugation. Such high fluorescence efficiencies of SMPI-TPA and SMPI-Cz confirmed that they could be efficient deep blue emitters. The calculated triplet energy levels of 2.32 eV (SMPI-TPA) and 2.40 eV (SMPI-Cz) are sufficiently high for the excitation of green dopants. The theoretically estimated singlet-to-triplet energy gaps (Δ*E*_ST_) of SMPI-TPA and SMPI-Cz are 0.36 eV and 0.30 eV, respectively, which are unfavourable for TADF emission. A small Δ*E*_ST_ value is required for efficient energy transfer from host T* (triplet excited state) to green phosphorescent emitters. The observed intense blue emission and high *T*_g_ of SMPI-TPA and SMPI-Cz confirmed their suitability as blue emitters. The device performances of the blue emitters were analysed by fabricating non-doped OLEDs with a configuration of ITO/NPB (50 nm)/TCTA (5 nm)/SMPI-TPA (I)/SMPI-Cz (II) (30 nm)/TPBI (15 nm)/LiF (1 nm)/Al (100 nm). It is clear from Fig. 4 and Table 1 that the as-fabricated novel SMPI-TPA- and SMPI-Cz-based blue OLEDs exhibit maximum brightness at low voltage. The blue EL and PL spectra of SMPI-TPA and SMPI-Cz in the solid state are similar (Fig. 3), and the hole injection barrier between SMPI-TPA and HTL (hole transport layer) is very small. Thus, effective electron–hole radiative recombination occurs in SMPI-TPA and SMPI-Cz layers. The small injection barrier of 0.28 (SMPI-TPA) and 0.32 eV (SMPI-Cz) for charge carriers accounts for the observed low turn-on voltages. The SMPI-TPA film exhibits smooth surface (roughness of 0.24 nm) and remains unchanged even after annealing (100 °C: 10 h) (Fig. 4: inset). The value of *η*_ex_ is calculated using the formula *η*_ex_ = *η*_out_ × *η*_rc_ × *η*_γ_ × *Φ*_PL_,^23^ where *η*_out_ is the light out-coupling efficiency (20%), *η*_rc_ is the product of the charge recombination efficiency (100%), *η*_γ_ is the efficiency of radiative exciton formation (25%) and *Φ*_PL_ is the photoluminescence quantum yield of emitters SMPI-TPA (0.56) and SMPI-Cz (0.71). Thus, the calculated *η*_ex_ values of SMPI-TPA- and SMPI-Cz-based devices are 2.8 and 3.6%, respectively. However, the obtained external quantum efficiencies (*η*_ex_) of SMPI-TPA- and SMPI-Cz-based devices are 6.1 and 5.9%, respectively, and the current efficiencies (*η*_c_) of SMPI-TPA- and SMPI-Cz-based devices are 5.3 and 5.1 cd A^−1^, respectively. The harvested *η*_ex_ exceeds the theoretical limit since delayed fluorescence is not expected and the obtained high *η*_ex_ is not in accordance with TADF.^25–30^ Time-resolved lifetime studies revealed monoexponential decay for SMPI-TPA and SMPI-Cz (Fig. S1†) with lifetime values in nanoscale, indicating the absence of delayed component (TADF) in emission. Both SMPI-TPA and SMPI-Cz do not possess delayed lifetime component. Only prompt species for SMPI-TPA (1.88 ns) and SMPI-Cz (1.92 ns) confirmed that SMPI-TPA and SMPI-Cz are different from TADF materials in luminous mechanism.^31^ The as-fabricated device structure ITO/NPB (50 nm)/TCTA (5 nm)/SMPI-TPA (I) or SMPI-Cz (II) (30 nm)/TPBI (15 nm)/LiF (1 nm)/Al (100 nm) shows that the TCTA layer plays a key role in achieving higher efficiency^32^ and the NPB layer was used as the hole-transporting layer (HTL) to be deposited in proximity to the SMPI-TPA and SMPI-Cz emissive layers. Due to the relatively small energy gap of NPB (*E*_g_ = −3.00 eV) in comparison to the energy gaps of the emissive layers SMPI-TPA (*E*_g_ = −3.26 eV) and SMPI-Cz (*E*_g_ = −3.22 eV), the excitons generated in the emissive layer are more likely to leak into HTL, leading to loss of excitons. However, herein, we used TCTA with *E*_g_ of −3.40 eV as a buffer layer to confine the excitons within the emissive layer, resulting in higher efficiency.^33^ Further, the EL spectra of the SMPI-TPA- and SMPI-Cz-based devices are identical to the corresponding PL emission of the thin film, implying balanced carrier transport and efficient confinement of excitons^33^ (Fig. 3). In the present study, some factors account for the efficiency of the devices: (i) bipolar carrier transporting properties of SMPI-TPA and SMPI-Cz, which contribute to better balance of carrier transport and wider distribution of recombination region within the emission layer (Fig. 3) and (ii) suitable HOMO and LUMO energies of SMPI-TPA and SMPI-Cz (Fig. 5); the hole injection barrier at TCTA : EML interface is 0.42 eV (SMPI-TPA) : 0.44 eV (SMPI-Cz) and electron injection barrier at TPBI : EML interface is 0.55 eV (SMPI-TPA) : 0.53 eV (SMPI-Cz). This reveals that there is only a small barrier for carrier injection, leading to high exciton formation even under high current density, resulting in higher device performances.^32,33^ The PL spectra of SMPI-TPA or SMPI-Cz were recorded in THF/water mixture with different water fractions (*f*_w_) to understand whether these materials show aggregation-induced emission. When a small amount of water (*f*_w_ = 10–90 vol%) was added to the THF solution of both SMPI-TPA and SMPI-Cz, the PL intensity remained unchanged, which shows that both SMPI-TPA and SMPI-Cz materials are AIE-inactive (Fig. S1†).

The small singlet–triplet splitting and good carrier transport properties allow both SMPI-TPA and SMPI-Cz to be used as hosts to fabricate red, green and yellow phosphorescence-emitting layers for PhOLEDs. The calculated PLQY of Ir(ppy)_3_-doped SMPI-TPA and Ir(ppy)_3_-doped SMPI-Cz was 0.61 and 0.79, respectively. In addition to high *η*_ex_ and *η*_c_, SMPI-TPA- and SMPI-Cz-based devices show high *η*_p_ (power efficiency) of 5.2 and 4.6 lm W^−1^, respectively. The blue emitters exhibit CIE coordinates of (0.16, 0.08 – SMPI-TPA) and (0.15, 0.07 – SMPI-Cz). The device based on SMPI-TPA shows maximum luminance of 12 680 cd m^−2^, and maximum current and power efficiencies of 5.3 cd A^−1^ and 5.2 lm W^−1^, respectively, at 3.1 V. These SMPI-TPA and SMPI-Cz compounds were also utilised as hosts for green dopants in the fabricated devices (Fig. 4) with a configuration of ITO/NPB (50 nm)/SMPI-TPA (30 nm):5 wt% Ir(PPy)_3_ or (III) or SMPI-Cz (30 nm):5 wt% Ir(PPy)_3_ (IV)/BCP (2,9-dimethyl-4,7-diphenyl-1,10-phenanthroline) (15 nm)/Alq_3_ (50 nm)/LiF (1 nm)/Al (100 nm). The energy level diagram of the as-fabricated device and molecular structures of materials used in the devices are shown in Fig. 5. Devices with SMPI-Cz (30 nm):5 wt% Ir(ppy)_3_ exhibit maximum luminance of 20 725 cd m^−2^, and *η*_c_ and *η*_p_ of 61.4 cd A^−1^ and 63.8 lm W^−1^, respectively, whereas SMPI-TPA:Ir(ppy)_3_-based devices exhibit high *η*_c_ and *η*_p_ values of 65.2 cd A^−1^ and 67.1 lm W^−1^, respectively. The maximum *η*_ex_ values of 19.6% and 23.4% were exhibited by SMPI-TPA:Ir(ppy)_3_ and SMPI-Cz:Ir(ppy)_3_, respectively. The lower efficiency from SMPI-TPA:Ir(ppy)_3_-based devices could be attributed to the lower triplet energy (*E*_T_ = 2.32 eV) of SMPI-TPA, which may cause back energy transfer from the guest triplet states to the host, resulting in loss of efficiency. The device performances reveal that SMPI-TPA and SMPI-Cz are universal hosts for green emitters. The EL spectra of devices III and IV were consistent with their corresponding PL spectra (Fig. 3), and no new peaks were observed under different operation voltages (2.8–10 V: Fig. S1†), implying that no excitons were wasted for host emission and effective exothermic energy transfer occurs from the host to the dopant in the emissive layer, which results in higher efficiency.^34,35^ In the present study, some factors account for the higher efficiency of the devices: (i) high triplet energies of 2.32 eV (SMPI-TPA) and 2.40 eV (SMPI-Cz) efficiently suppress the energy return from the dopant to the host, resulting in higher efficiency and (ii) good thermal stability restrains the strong bimolecular interaction of the phosphorescent emitters to reduce the triplet–triplet annihilation.^34^ These results indicate that the introduction of a bipolar molecule is a practical strategy for achieving highly efficient OLEDs both as a blue emitter and as a host for green emission.

## 4. Conclusion

In this study, we have reported the newly fabricated efficient deep blue-emissive materials with balanced transport and high thermal properties. Non-doped blue emitters with SMPI-TPA and SMPI-Cz exhibit higher electroluminescent efficiencies. Devices with SMPI-TPA show maximum luminance of 12 680 cd m^−2^ and maximum current (*η*_c_) and power (*η*_p_) efficiencies of 5.3 cd A^−1^ and 5.2 lm W^−1^, respectively, at 3.1 V. The device with configuration of SMPI-Cz (30 nm):5 wt% Ir(ppy)_3_ exhibits maximum luminance of 20 725 cd m^−2^, whereas the *η*_c_ and *η*_p_ exhibited by the SMPI-TPA:Ir(ppy)_3_ device are 65.2 cd A^−1^ and 67.1 lm W^−1^, respectively. The maximum *η*_ex_ of 19.6% and 23.4% were exhibited by SMPI-TPA:Ir(ppy)_3_- and SMPI-Cz:Ir(ppy)_3_-based devices, respectively. The lower efficiency from SMPI-TPA:Ir(ppy)_3_-based devices could be attributed to the lower triplet energy (*E*_T_ = 2.32 eV) of SMPI-TPA, which may cause back energy transfer from the guest triplet states to the host, resulting in loss of efficiency. The device performance indicates that introduction of a bipolar molecule is a practical strategy for achieving efficient OLEDs both as a blue emitter and as a host for green emission.

## Conflicts of interest

There are no conflicts to declare.

## Supplementary Material

RA-008-C8RA02840K-s001
