# Supplementary material for: Efficient donor–acceptor host materials for green organic light-emitting devices: non-doped blue-emissive materials with dual charge transport properties
Source: RSC Adv. 2018 May 31;8(36):20007–15. doi: 10.1039/c8ra02840k (PMC9080778; doi:10.1039/c8ra02840k)
Supplement: RA-008-C8RA02840K-s001 [file RA-008-C8RA02840K-s001.pdf]

Electronic Supplementary Material (ESI) for RSC Advances.
This journal is © The Royal Society of Chemistry 2018

Supporting Information

Efficient donor–acceptor host materials for green organic light emitting devices: Non-doped blue emissive materials with dual charge transport properties

Jayaraman Jayabharathi*, Palanisamy Sujatha, Venugopal Thanikachalam, Pavadai Nethaji

Department of Chemistry, Annamalai University, Annamalainagar 608 002, Tamilnadu, India

* Tel: +91 9443940735; *E-mail address:* jtchalam2005@yahoo.co.in

Scheme S1: Synthetic route of SMPI-TPA and SMPI-Cz

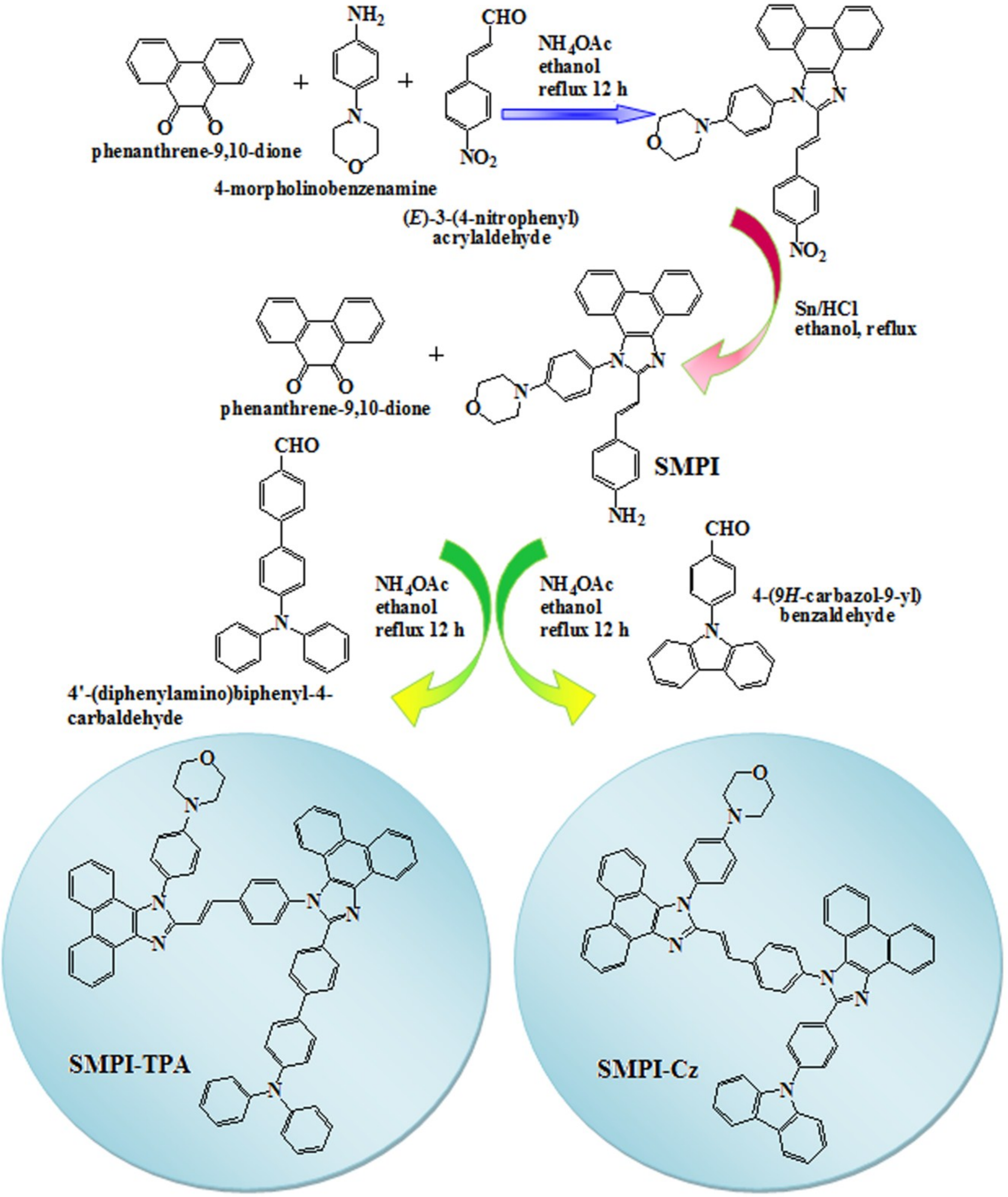

Figure S1: (a) Lifetime spectra of SMPI-TPA and SMPI-Cz; (b) EL spectra of devices at different voltages; (c) PL spectra of SMPI-TPA and SMPI-Cz in various THF/Water fractions

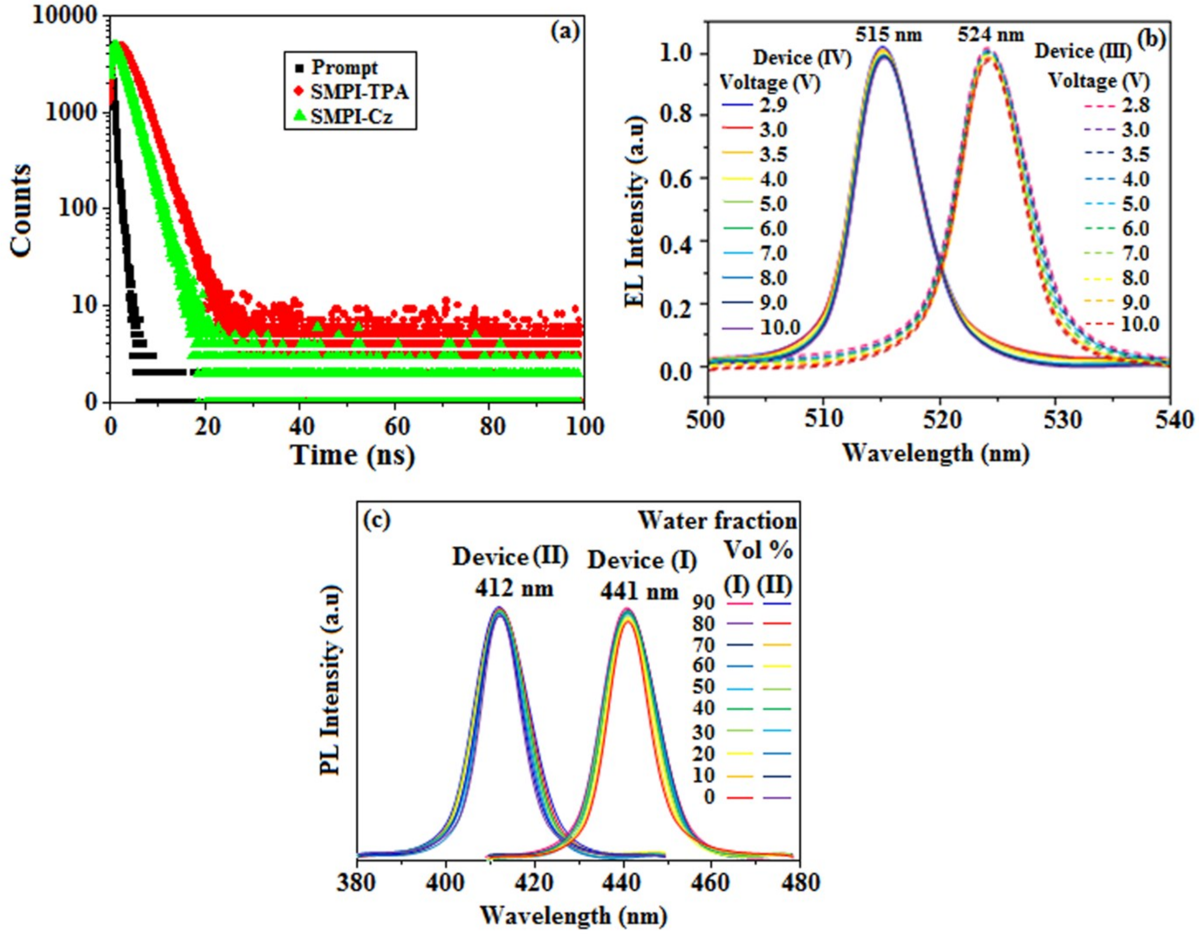

Table S1 (a). Computed vertical transitions, oscillator strengths and configurations of SMPI-Cz

Compound	DCM		
	λ_{max} (nm)	*F*	Configuration
SMPI-Cz	405.62	3.0566	HOMO→LUMO+1 (-0.10947)
	385.88	3.2130	HOMO→LUMO+1 (-0.24067)<br>HOMO→LUMO+2 (-0.18243)
	356.28	3.4800	HOMO→LUMO+3 (0.34985)<br>HOMO→LUMO+4 (0.13568)

Excitation energies and oscillator strengths:

Excited State 1: Singlet-A 3.0566 eV 405.62 nm f=0.8785
186 ->188 0.20644
186 ->189 -0.10947
187 ->188 0.57175
187 ->194 0.14396
187 ->202 -0.10687
This state for optimization and/or second-order correction.
Copying the excited state density for this state as the 1-particle RhoCI density.
Excited State 2: Singlet-A 3.2130 eV 385.88 nm f=0.9619
185 ->190 0.11239
186 ->188 0.15216
186 ->189 0.48271
186 ->190 -0.18243
186 ->193 -0.19009
187 ->189 -0.24067
Excited State 3: Singlet-A 3.4800 eV 356.28 nm f=0.0776
183 ->188 -0.16745
183 ->191 -0.20707
183 ->194 -0.14688
186 ->191 0.17621
186 ->194 -0.17345
187 ->191 0.34985
187 ->192 0.13568
187 ->193 0.14815
187 ->194 -0.30833
Excited State 4: Singlet-A 3.4900 eV 355.25 nm f=0.0703
182 ->189 -0.15026
182 ->190 0.22447
182 ->193 0.10898
186 ->190 -0.21055
186 ->192 0.29475
186 ->193 0.32962
187 ->190 0.10493
187 ->191 0.16089

187 ->193 -0.11489
187 ->194 -0.11491
Excited State 5: Singlet-A 3.6704 eV 337.80 nm f=0.3700
180 ->189 -0.10152
182 ->192 -0.10574
182 ->193 -0.12308
185 ->189 0.28369
185 ->190 0.10116
185 ->192 0.11466
185 ->200 -0.11822
186 ->190 0.33154
186 ->192 -0.10343
186 ->193 0.13781
186 ->200 0.12125
187 ->190 -0.11819
187 ->191 0.16920
187 ->194 0.11887

Table S1 (b). Computed vertical transitions, oscillator strengths and configurations of SMPI-TPA

Compound	DCM		
	λ_{max} (nm)	*F*	Configuration
SMPI-TPA	404.79	3.0629	HOMO→LUMO+1 (-0.30391)
	353.01	3.5122	HOMO→LUMO+1 (-0.13435)<br>HOMO→LUMO+2 (-0.13515)
	343.28	3.6117	HOMO→LUMO+3 (-0.13404)

Excitation energies and oscillator strengths:
Excited State 1: Singlet-A 3.0272 eV 409.57 nm f=0.5821
187 ->189 0.37240
187 ->191 0.12311
188 ->189 0.41402
188 ->190 0.27685
This state for optimization and/or second-order correction.
Copying the excited state density for this state as the 1-particle RhoCI density.
Excited State 2: Singlet-A 3.0629 eV 404.79 nm f=0.4771
187 ->189 -0.16852
187 ->190 0.43703
187 ->192 -0.10274
188 ->189 0.28275
188 ->190 -0.30391
Excited State 3: Singlet-A 3.5122 eV 353.01 nm f=0.0388
184 ->190 0.13088
185 ->189 0.23060

186 ->189 0.38708
186 ->190 -0.30889
187 ->190 -0.10493
188 ->190 -0.13435
188 ->191 -0.13515
Excited State 4: Singlet-A 3.6117 eV 343.28 nm f=0.2915
182 ->190 -0.10104
183 ->191 0.10394
186 ->190 0.11761
187 ->191 0.13995
187 ->192 0.25731
187 ->194 0.16139
187 ->196 -0.26785
188 ->191 -0.23528
188 ->192 -0.13404
188 ->194 -0.11659
188 ->195 0.14398
188 ->196 0.20125
Excited State 5: Singlet-A 3.6230 eV 342.21 nm f=0.1207
182 ->189 0.13254
182 ->191 0.18393
187 ->191 0.21306
187 ->195 -0.27564
188 ->191 0.15815
188 ->192 -0.20211
188 ->193 -0.15121
188 ->195 -0.27078
188 ->196 0.14523
